# *Pectobacterium atrosepticum* KDPG aldolase, Eda, participates in the Entner–Doudoroff pathway and independently inhibits expression of virulence determinants

**DOI:** 10.1111/mpp.13025

**Published:** 2020-12-10

**Authors:** Huan Wang, Yujie Wang, Sonia Humphris, Weihua Nie, Pengfei Zhang, Frank Wright, Emma Campbell, Baishi Hu, Jiaqin Fan, Ian Toth

**Affiliations:** ^1^ Department of Plant Pathology Nanjing Agricultural University Nanjing China; ^2^ Cell and Molecular Science James Hutton Institute Dundee UK; ^3^ Institute of Agricultural Science of Taihu Lake District Suzhou China; ^4^ Bioinformatics and Statistics James Hutton Institute Dundee UK

**Keywords:** Eda, Entner–Doudoroff pathway, pathogenicity, *Pectobacterium*

## Abstract

*Pectobacterium carotovorum* has an incomplete Entner–Doudoroff (ED) pathway, including enzyme 2‐keto‐3‐deoxy‐6‐phosphogluconate aldolase (Eda) but lacking phosphogluconate dehydratase (Edd), while *P. atrosepticum* (Pba) has a complete pathway. To understand the role of the ED pathway in *Pectobacterium* infection, mutants of these two key enzymes, Δ*eda* and Δ*edd*, were constructed in Pba SCRI1039. Δ*eda* exhibited significant decreased virulence on potato tubers and colonization in planta and was greatly attenuated in pectinase activity and the ability to use pectin breakdown products, including polygalacturonic acid (PGA) and galacturonic acid. These reduced phenotypes were restored following complementation with an external vector expressing *eda*. Quantitative reverse transcription PCR analysis revealed that expression of the pectinase genes *pelA*, *pelC*, *pehN*, *pelW*, and *pmeB* in Δ*eda* cultured in pyruvate, with or without PGA, was significantly reduced compared to the wild type, while genes for virulence regulators (*kdgR*, *hexR*, *hexA*, and *rsmA*) remained unchanged. However, Δ*edd* showed similar phenotypes to the wild type. To our knowledge, this is the first demonstration that disruption of *eda* has a feedback effect on inhibiting pectin degradation and that Eda is involved in building the arsenal of pectinases needed during infection by *Pectobacterium*.

## Graphical abstract

A component of the Entner–Doudoroff pathway, 2‐keto‐3‐deoxy‐6‐phosphogluconate aldolase, plays a vital role in the virulence of *Pectobacterium atrosepticum* on potato by controlling expression of genes involved in pectin degradation.

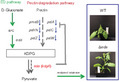

## 1 INTRODUCTION

**FIGURE 1:**
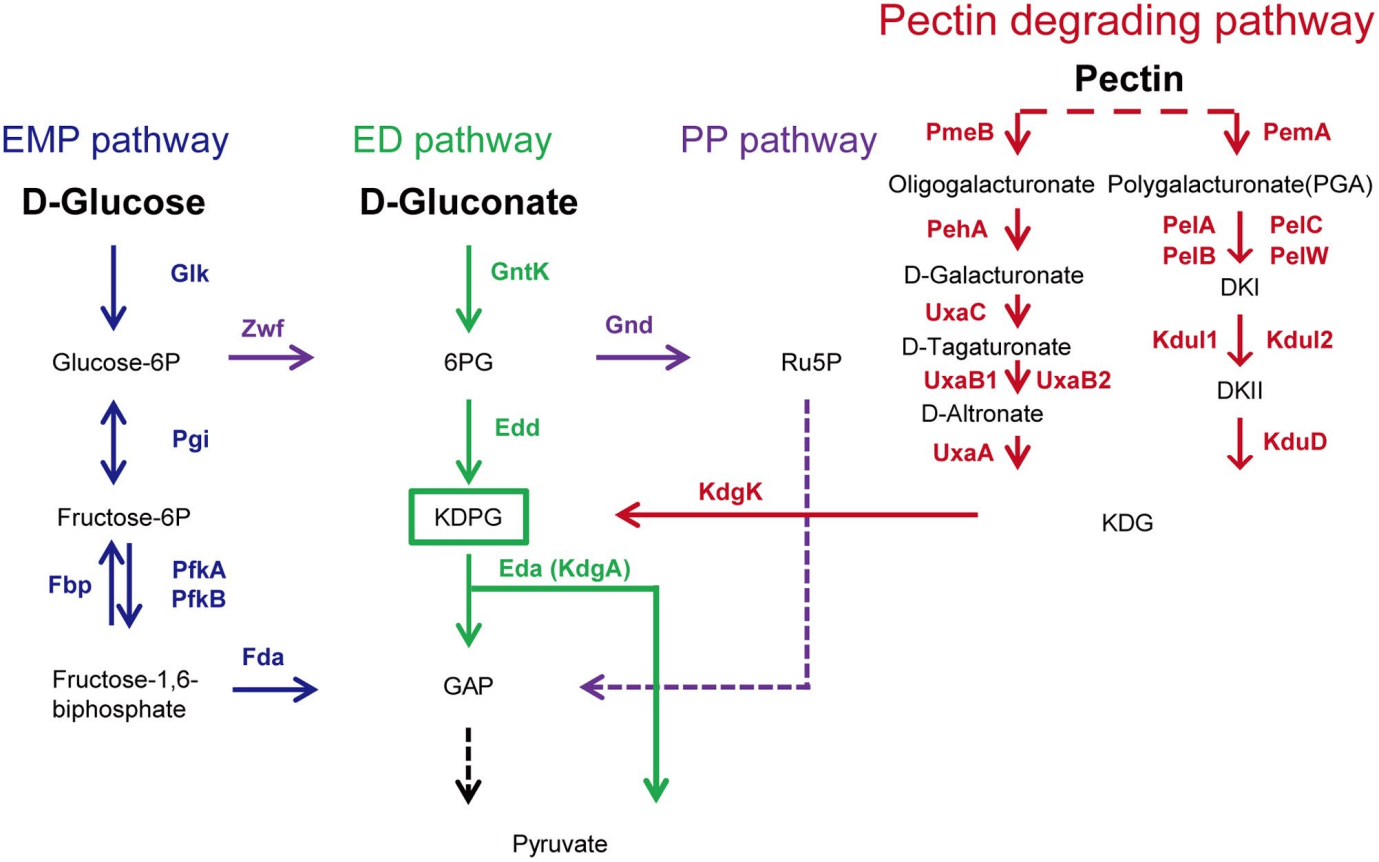
Major glycolytic pathways in *Pectobacterium atrosepticum*. Different metabolic pathways are displayed in different colours. Solid arrows represent reactions that are catalysed by a single enzyme, while dashed arrows indicate multiple reactions. The key products are boxed. The pectin degrading pathway is presented in red; EMP, Embden–Meyerhof–Parnas pathway (blue); ED, Entner–Doudoroff pathway (green); PP, pentose phosphate pathway (purple); 6PG, 6‐gluconate‐phosphate; Ru5P, ribulose 5‐phosphate; DKI, 5‐keto‐4‐deoxyuronate; DKII, 2,5‐diketo‐3‐deoxygluconate; KDG, 2‐keto‐3‐deoxygluconate; KDPG, 2‐keto‐3‐deoxy‐6‐phosphate‐gluconate; GAP, glyceraldehyde‐3‐phosphate

The Entner–Doudoroff (ED) pathway is one of the central glycolytic pathways in gram‐negative bacteria (Figure 1; Conway, 1992). It was first described in *Pseudomonas saccharophila* (Entner & Doudoroff, 1952) and was later discovered in a wide range of organisms from *Archaea* to plants (Flamholz et al., 2013). Although the ED pathway produces only one adenosine triphosphate (ATP) per glucose molecule, which is half that of the Embden–Meyerhof–Parnas (EMP) pathway, it has the advantage of requiring several‐fold less enzymatic protein to achieve the same glucose conversion rate. Furthermore, genomic analysis has revealed that the EMP pathway is the major choice for energy‐deprived anaerobes as it has a higher ATP yield, whereas the ED pathway is preferred by facultative anaerobes and aerobes (Flamholz et al., 2013).

The ED pathway has two unique enzymes, 6‐phosphogluconate dehydratase (Edd, EC 4.2.1.12) and 2‐keto‐3‐deoxy‐6‐phosphogluconate (KDPG) aldolase (Eda, EC 4.1.2.14). 6‐phosphogluconate is dehydrated by Edd to form KDPG, which is then cleaved by Eda to form pyruvate and glyceraldehyde 3‐phosphate (GAP). The primary function of the ED pathway is the breakdown of sugar acids such as gluconate, which cannot be metabolized through the EMP pathway (glucose metabolism) (Peekhaus & Conway, 1998). Gluconate is a major carbon source used by *Escherichia coli* MG1655 in the colonization of the mouse intestine. It was found that a deletion within *edd* negatively affects both disease initiation and pathogen maintenance within the host (Chang et al., 2004). Transcriptional activation analyses and gene silencing revealed that in *Vibrio cholerae* the ED pathway is obligatory for gluconate utilization and regulating virulence (Patra et al., 2012). A recent study also suggested that nicotinamide adenine dinucleotide phosphate, generated by the ED pathway, is required for counteracting oxidative stress in *Pseudomonas putida* KT2440, which consumes glucose almost exclusively through the ED pathway (Chavarria et al., 2013). In addition to these virulence‐related roles, the ED pathway is also critical to the survival of the pathogenic organisms *Legionella pneumophila* and *Shigella flexneri* in their host systems (Harada et al., 2010; Waligora et al., 2014). However, the role of the ED pathway in virulence is not conserved in all pathogenic organisms as it has a limited impact on *Helicobacter pylori* colonization of mice (Wanken et al., 2003).

Gram‐negative bacterial species within the *Pectobacterium* genus are listed in the top 10 most important plant‐pathogenic bacteria, causing blackleg and soft rot on potato and diseases on many other crops and ornamental plants (Mansfield et al., 2012). *Pectobacterium* spp. employ a series of plant cell wall‐degrading enzymes (PCWDEs), which are mainly secreted through the Type II secretion system (T2SS), to break down the host plant cell wall (Toth et al., 2003). These are the main virulence determinants in *Pectobacterium* spp. but, in addition to the PCWDEs, small virulence proteins such as Nip and Svx, flagella and the Type III (T3SS) and Type VI (T6SS) secretion systems are also involved (Corbett et al., 2005; Holeva et al., 2004; Laasik et al., 2014; Liu et al., 2008; Mattinen et al., 2004). The regulation of these factors is controlled by a complex network of regulators, including the quorum‐sensing system (e.g., ExpI/ExpR), two‐component systems (e.g., GacA/GacR), LysR family regulators (e.g., KdgR, RexZ), and a small RNA system (RsmA/RsmB/RsmC) (Babujee et al., 2012; Barnard & Salmond, 2007; Faure & Dessaux, 2007; Liu et al., 2008; Lyon, 1989; Toth et al., 2006, 2015). Environmental factors such as oxygen, host plant extracts, and divalent cations have also been reported to affect virulence in *Pectobacterium* (Babujee et al., 2012; Flego et al., 1997; Mattinen et al., 2007). In *Dickeya* spp. (formerly *Erwinia chrysanthemi*) Eda (referred to as KdgA) was shown to be induced by pectin‐degrading products in vivo through the action of the negative regulator KdgR. However, no role in virulence was established (Hugouvieux‐Cotte‐Pattat & Robert‐Baudouy, 1994). Recently, the *vgu* operon involved in gluconate metabolism was reported to be required for correct expression of virulence through regulators KdgR, FlhD, HexA, and RsmA in *P. carotovorum* WPP14 (Mole et al., 2010). However, as results were based on a multigene deletion, and the WPP14 strain lacks the *edd* gene, the role of the ED pathway (including genes *edd* and *eda*) in these plant‐pathogenic organisms remains unclear.

In our previous work, we reported that the KDPG adolase (Eda) in the ED pathway is required for full virulence of *P. carotovorum* strain PccS1 (Wang et al., 2018). However, details of the role of Eda or the ED pathway in virulence were not determined. In this study, we conducted a full functional analysis of the ED pathway key enzymes (Edd and Eda) in the virulence of *P. atrosepticum*. We identified that, while the ED pathway is not directly involved in virulence of *Pectobacterium*, Eda is through a role in pectin degradation controlled through the expression of PCWDEs.

## 2 RESULTS

### 2.1 A complete ED pathway exists in *P. atrosepticum*, *P. parmentieri*, and *P. wasabiae* but not in *P. carotovorum* or other *Pectobacterium* species and is critical for gluconate utilization

**FIGURE 2:**
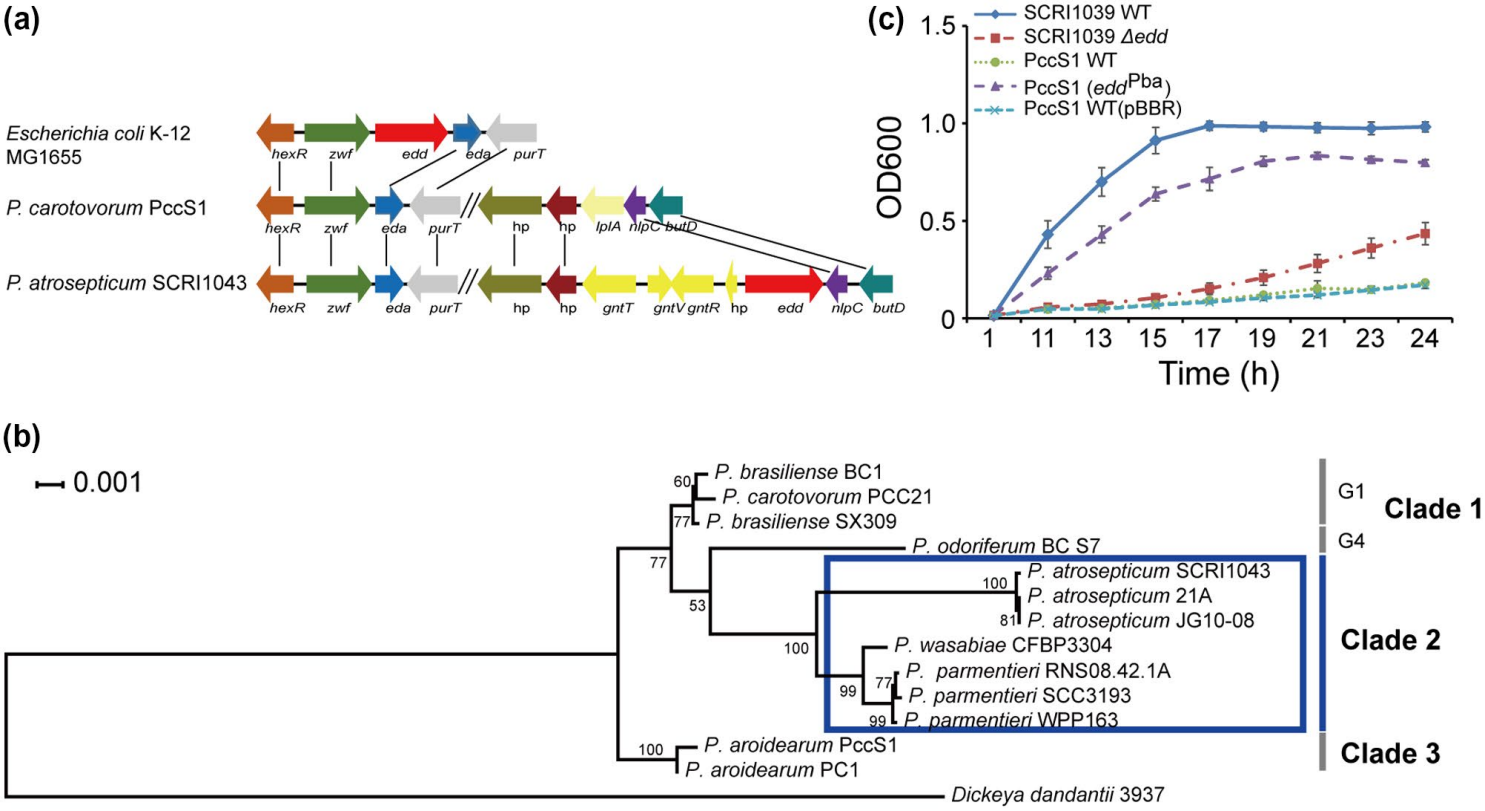
Gene *edd* is essential for *Pectobacterium* in utilization of gluconate. (a) Operon structure of *edd* and *eda* in different bacterial species. (b) Phylogenetic analysis of 13 *Pectobacterium* strains revealed a possible role in host adaption for the Entner–Doudoroff pathway. Protein sequences of 31 housekeeping genes from 14 bacterial genomes were extracted. *Dickeya dadantii*3937 was used as an outgroup for the analysis. A RAxML tree was developed using the IQ‐TREE web server with partitions. The blue rectangle indicates that the *edd* gene is present in these genomes. The bootstrap value was inferred from 1,000 replicates. (c) Growth curve over 24 hr in minimal media using gluconate as the sole carbon source of *P. carotovorum* PccS1 (which lacks gene *edd*) and following complementation with the *edd* gene (*edd* ^Pba^) from *P. atrosepticum* SCRI1039 together with the SCRI1039 wild type and ∆*edd* mutant strain. Values are the means ± *SE* of three independent experiments

In a previous study, we reported that a ∆*eda* mutant of *P. carotovorum* PccS1 was significantly attenuated in virulence on *Zantedeschia elliottiana*. However, bacterial growth in both Luria‐Bertani (LB) and minimal medium (MM) supplemented with 0.2% glucose (MM + 0.2% glucose) was not affected by the deletion (Wang et al., 2018). To explore the role of *eda* in the metabolism pathways, we performed a genomic comparison between *P. carotovorum* PccS1 and related pathogenic bacteria *P. atrosepticum* and *E. coli*. The results (Figure 2a) show that the genome of PccS1 carries the *eda* but not the *edd* gene, while *P. atrosepticum* (Pba) SCRI1043 and *E. coli* K‐12 carry both *eda* and *edd* genes, which are jointly necessary for the complete ED pathway (Conway, 1992).

We conducted a phylogenetic analysis to further demonstrate the potential role of the ED pathway in *Pectobacterium* species. Based on the protein sequences of 31 housekeeping genes, a total of 13 *Pectobacterium* strains were used to conduct a phylogeny inference with *Dickeya dadantii* 3937 set as an outgroup. A rectangular phylogram shows that these *Pectobacterium* strains are generally subdivided into three clades (Figure 2b). Clade 1 includes *P. carotovorum* PCC21, *P. brasiliense* strains BC1 and BX309, and *P. odoriferum* BC S7. When compared with the phylogenetic tree obtained by Zhang et al. (2016), which showed similar groupings for these strains, Clade 1 can be subdivided into two groups: BC1, PCC21, and SX309 were classified into group 1 (G1), and BC S7 into group 4 (G4). The species in Clade 1 lack the *edd* gene and therefore do not have a complete ED pathway. Clade 2 includes three species, *P. atrosepticum* (three strains), *P. wasabiae* (one strain), and *P. parmentieri* (Ppa, three strains), all of which possess all components of the ED pathway, and Clade 2 forms an evolutionary group distinct from other *Pectobacterium* species (Clade 1 and Clade 3), which all lack the *edd* gene. Clade 3 includes two strains: PccS1, isolated from rotted *Zantedeschia elliottiana* (calla lily) in China, shared a high similarity with PC1 isolated from *Ornithogalum dubium* in Israel.

Although PccS1 could not utilize d‐gluconate, it could do so when the gene *edd* ^Pba^ was introduced into the strain (Figure 2c). The results therefore indicate that *edd* ^Pba^ was successfully expressed heterogeneously and confirms that PccS1 has an incomplete ED pathway. Moreover, a mutant of Pba SCRI1039 lacking *edd* showed attenuated growth in MM supplemented with gluconate at 0.2% as the sole carbon source compared with the wild‐type SCRI1039 (Figure 2c). The results further demonstrate that a completed ED pathway is necessary for the utilization of gluconate, which only Clade 2 isolates are able to undertake.

### 2.2 Mutant ∆*eda* shows significantly reduced pathogenicity by *P. atrosepticum* SCRI1039 on potato tubers, while mutant Δ*edd* has no observable effect

**FIGURE 3:**
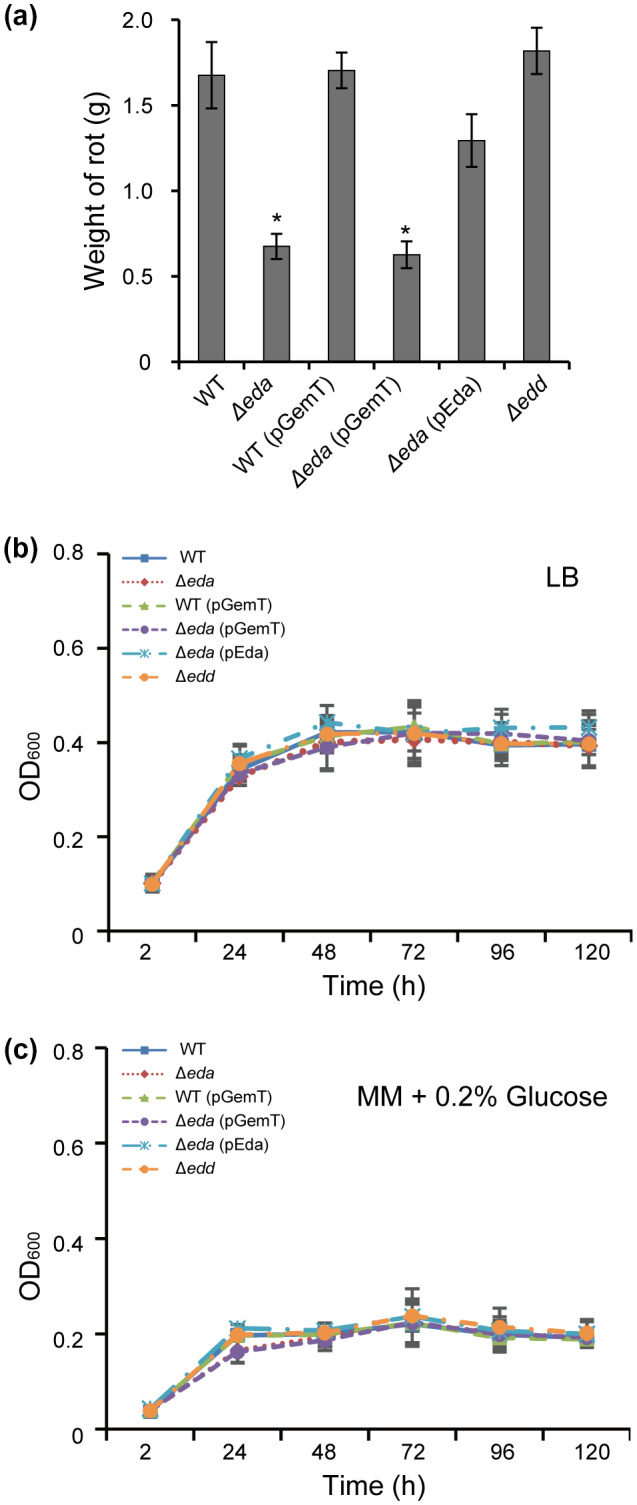
Mutant Δ*eda* is defective in virulence on potato tubers. (a) Tuber slides were inoculated with Δ*eda* and Δ*edd* strains derived from *Pectobacterium atrosepticum* SCRI1039 (WT). The *y* axis shows grams of macerated tissues measured 72 hr after inoculation. The experiment had six internal replicates and was conducted on at least three occasions. Values are the means ± *SE*. *Significant difference between wild type and mutant (*p* < .05; Duncan's multiple range test). (b) and (c) Mutants Δ*eda* and Δ*edd* had no effect on growth in media. Growth curves of *P. atrosepticum* SCRI1039 in Luria‐Bertani (LB) (b) and minimal medium (MM) supplemented with 0.2% glucose (c) over 120 hr. Values are the means ± *SE* of three independent experiments

To better understand the role of the key components of the ED pathway during infection of *P. atrosepticum*, two mutants, ∆*eda* and ∆*edd*, were constructed in Pba SCRI1039. The mutants were confirmed by PCR and sequencing of *eda* and *edd* fragments. To determine whether *eda* and *edd* are involved in virulence, we first compared tuber maceration by ∆*eda* and ∆*edd* mutants with that of the wild type. Each potato tuber slice was inoculated with a single strain using 10 mM MgSO_4_ buffer as a negative control. The wild type, wild type with empty pGemT vector, and Δ*edd* mutant all macerated the tuber slices to a similar extent over a 3‐day period, which indicates that the *edd* gene had no observable effect on virulence. However, mutant Δ*eda* and Δ*eda* with the empty vector showed a lower maceration level on the potato tuber slices than the wild type (Figure 3a). ∆*eda*(pEda), which carried the complementing fragment of *eda*, restored the mutant's ability to macerate the tuber slices, although it did not reach wild‐type levels (Figure 3a).

When cultured in LB medium and MM plus 0.2% glucose, Δ*eda* grew at an equivalent level to the wild‐type strain over a 5‐day period (Figure 3b,c). This suggested that the attenuated virulence in Δ*eda* was not due to its ability to grow using simple sugars.

### 2.3 *P. atrosepticum* SCRI1039 ∆*eda* shows significantly attenuated virulence and colonization on potato stems

**FIGURE 4:**
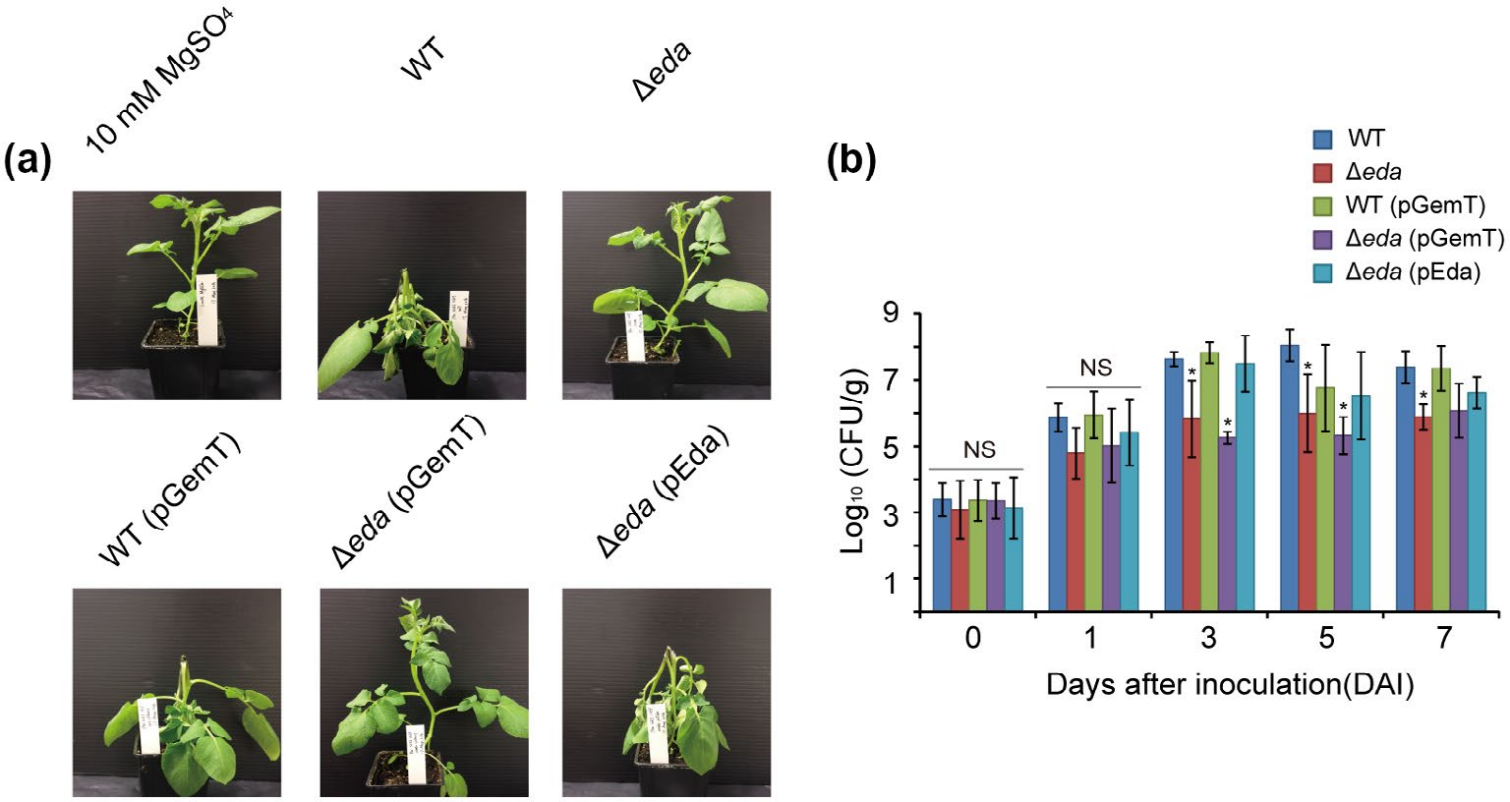
Mutant Δ*eda* did not cause typical blackleg symptoms on potato plants and was reduced in its ability to colonize in planta. (a) Plants were inoculated with 10^5^ cfu/ml Δ*eda* derived from *Pectobacterium atrosepticum* SCRI1039 (WT) using 10 mM MgSO_4_ as a negative control. Images were collected at 3 days after inoculation when typical blackleg symptoms on potato (cv. Estima) appeared. (b) Infected plants were ground and bacteria extracted on the day of inoculation and 1, 3, 5, and 7 days later. Colonies were counted on crystal violet pectate medium. Each strain was inoculated into three plants and the experiment was repeated twice. Values are the means ± *SE* of log_10_cfu per gram plant tissue from three independent experiments

To determine whether ∆*eda* was reduced in virulence on potato plants as well as tubers, the ability of the wild type and Δ*eda* to colonize and cause disease on potato stems was assayed (Figure 4). Unlike the wild‐type strain, Δ*eda* was unable to develop blackleg symptoms 3 days postinfiltration (Figure 4a). The population of the wild‐type strain in the stems increased from c.10^3^ to 10^8^ cfu/g during this period, showing a slight decline by day 7 (Figure 4b). Δ*eda*, on the other hand, was 2 logs lower at c.10^6^ cfu/g by day 3 and the population remained at that level up to day 7 (Figure 4b). Following complementation, both blackleg symptoms and a population level equivalent to that of the wild type were observed in Δ*eda* (Figure 4a,b).

### 2.4 Mutant ∆*eda* is attenuated in pectin degradation

**FIGURE 5:**
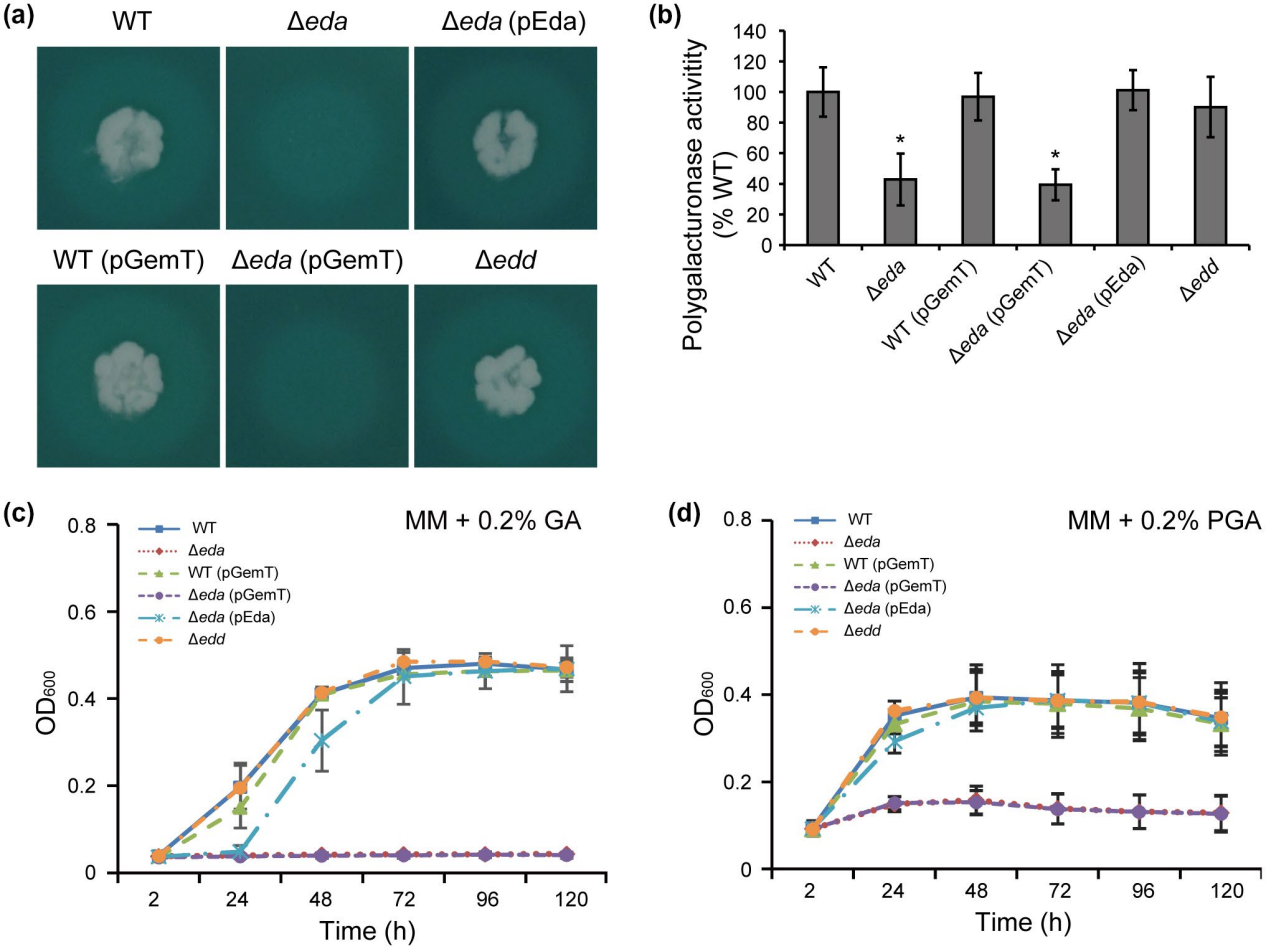
Mutant Δ*eda* of *Pectobacterium atrosepticum* SCRI1039 is deficient in pectin degradation. The activities of pectinase (a) in *P.* *atrosepticum* SCRI1039 wild type (WT), ∆*eda*, and ∆*edd* were carried out on agar plates. Diameters of haloes around the colonies were calculated and statistically analysed (b). The *y* axis represents means of percentage compare to the WT (%) ± *SE* from three independent experiments. *Significant difference between the WT and mutant strains (*p* < .05; Duncan's multiple range test); NS, no significant difference (*p* < .05; Duncan's multiple range test). Bacterial growth in minimal medium (MM) supplemented with pectin degradation products (c) galacturonic acid (GA) and (d) polygalacturonic acid (PGA) were evaluated over 120 hr. Values are the means ± *SE* of three independent experiments

It has been demonstrated that PCWDEs are the main virulence factors of *Pectobacterium* spp. and are a crucial prerequisite during plant infection and subsequent disease development (Toth et al., 2003). Recently, we reported that the ∆*eda* mutant of *Pectobacterium carotovorum* PccS1 was significantly reduced in polygalacturonase activity on pectin agar plates (Wang et al., 2018). A similar result was observed when the Pba SCRI1039 ∆*eda* mutant was patched onto the assay plates (Figure 5a,b), while cellulase activity was unaffected compared with the wild type (Figure S1a,b) and neither of these two assays showed reduced activity in the ∆*edd* mutant.

We further analysed the abilities of SCRI1039 ∆*eda* and ∆*edd* to utilize pectin breakdown products by determining their growth ability in MM supplemented with polygalacturonic acid (PGA) or galacturonic acid (GA). When these were used as a sole carbon source supplemented in MM, ∆*eda* did not grow or grew to a very low level on PGA and GA, respectively (Figure 5c,d). Expression of the *eda* gene in trans in ∆*eda*(pEda) fully restored polygalacturonase activity and the ability to grow (Figure 5a–d), which suggests that the decreased ability to grow in media and attenuated virulence on the host was due to the reduced ability of ∆*eda* to metabolize the breakdown products of pectin. The ∆*edd* mutant, however, showed no observable changes in enzyme activities or pectin utilization compared to the wild type (Figure 5a–d). The results indicate that Edd does not participate in pectin utilization in *P. atrosepticum* SCRI1039, whereas Eda is crucial in this role.

### 2.5 Mutant ∆*eda* exhibits changes in expression of genes encoding enzymes for pectin degradation

**FIGURE 6:**
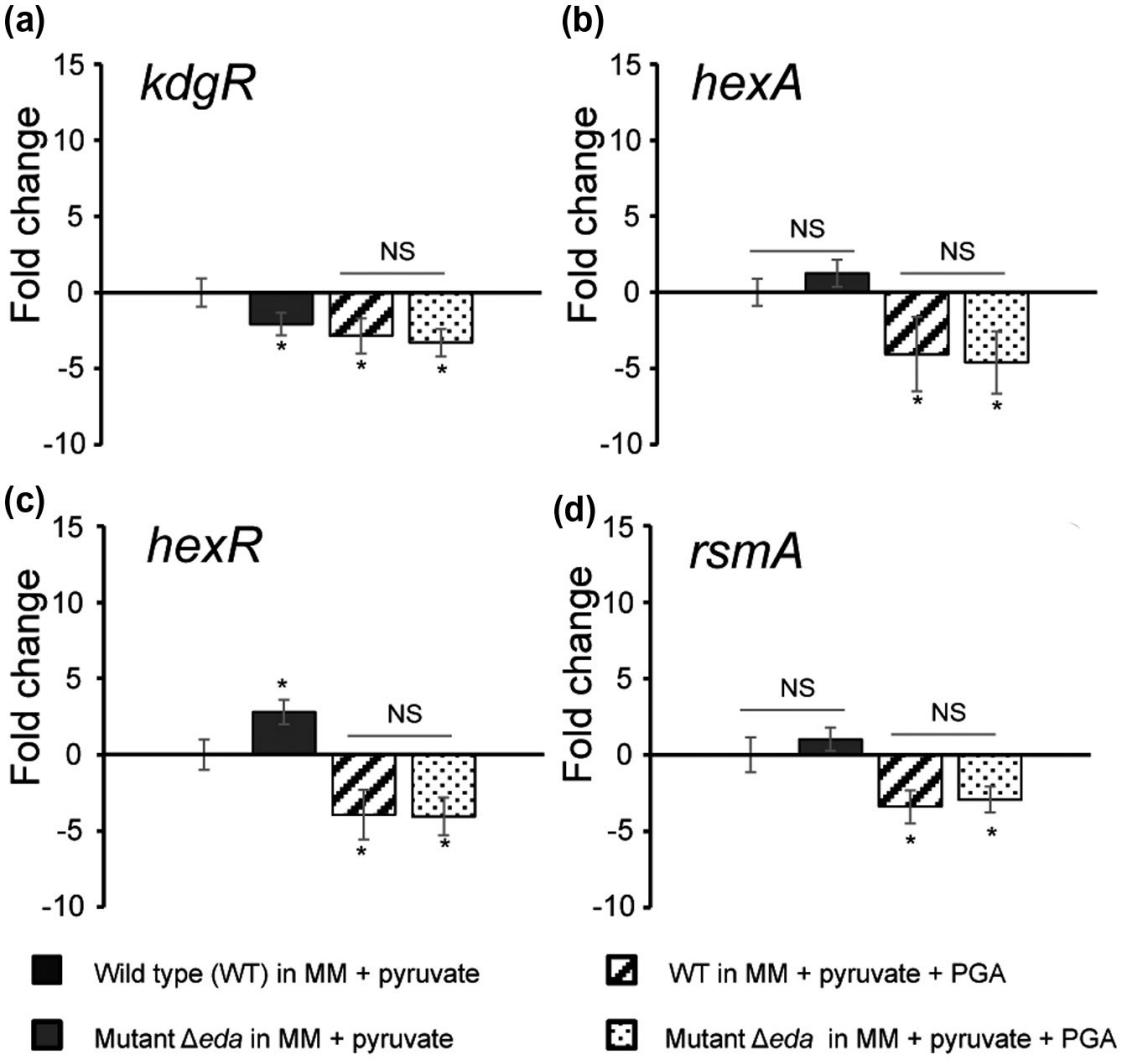
Relative transcript levels of regulators were measured in *Pectobacterium atrosepticum* strains wild type (WT) and ∆*eda. P. atrosepticum* SCRI1039 WT and ∆*eda* mutant were grown at 28 °C under different culture conditions (minimal medium [MM] plus 0.2% pyruvate and supplemented with or without polygalacturonic acid [PGA]) to OD_600_ 0.4–0.6. The *recA* gene was used as a control housekeeping gene. Transcript levels of wild type in pyruvate were set as zero and the other three treatments were normalized to the wild type. The expression levels of up‐regulated genes were positive while those of down‐regulated genes were negative (a)–(f). Error bars represent standard errors for triplicate assays. *p < .05, **p < .01, NS, no significant difference, Duncan's multiple range test

To understand the role of the ED pathway key enzyme KDPG adolase (Eda) in virulence and PCWDE activity in *P. atrosepticum*, a quantitative reverse transcription PCR (RT‐qPCR) analysis was conducted to determine whether the expression levels of genes encoding regulators of virulence and carbon metabolism (*kdgR, hexA, hexR*, and *rsmA*) were affected in ∆*eda* compared to that of the wild type. When pyruvate (the ultimate breakdown product of both the ED pathway and pectin degradation) was used as the sole carbon source, ∆*eda* exhibited similar RNA levels to that of the wild type for most of the regulators tested, and comparable levels were also seen for the wild type and ∆*eda* in the presence of PGA (Figure 6a–d). However, gene expression for both strains was significantly down‐regulated for all four regulators when grown in PGA compared to pyruvate (Figure 6a–d). This suggests that reduced growth on pectin agar plates was not due to the influence of the *eda* gene on the expression of these virulence regulators.

**FIGURE 7:**
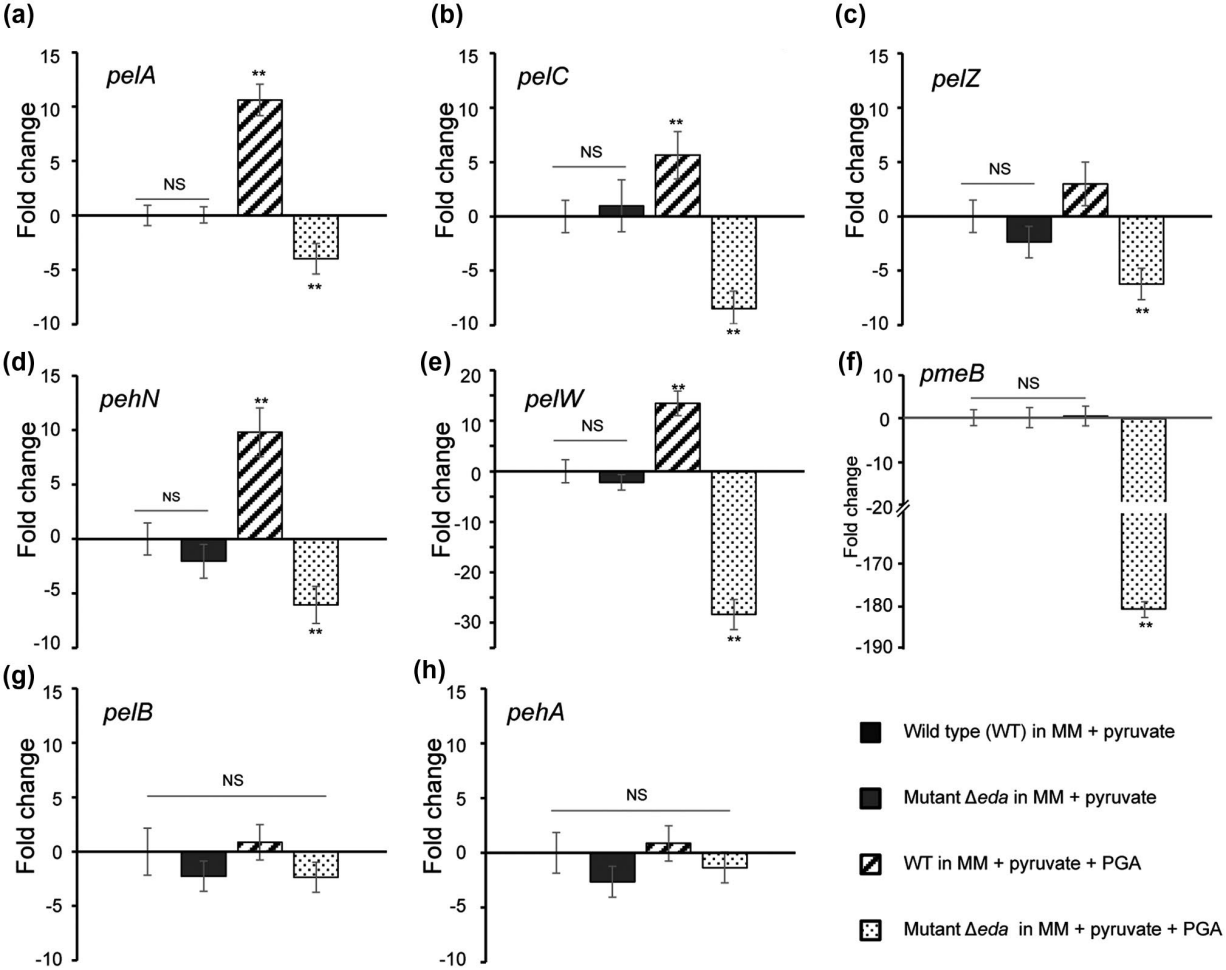
Relative transcript levels of selected genes were measured in *Pectobacterium atrosepticum* strains wild type (WT) and ∆*eda. P. atrosepticum* SCRI1039 WT and ∆*eda* mutant were grown at 28 °C under different culture conditions (minimal medium [MM] plus 0.2% pyruvate and supplemented with or without polygalacturonic acid [PGA]) to OD_600_ 0.4–0.6. The *recA* gene was used as a control housekeeping gene. Transcript levels of WT in pyruvate were set as zero and the other three treatments were normalized to the WT. The expression levels of up‐regulated genes were positive while those of down‐regulated genes were negative (a)–(l). Error bars represent *SE* for triplicate assays. Difference between the treatment and WT in pyruvate condition, Duncan's multiple range test, *p < .05; **p < .01; NS, no significant difference *p* > .05

To further determine whether reduced pectinase activity observed on pectin agar plates (Figure 5a,b) was due to changes in the expression of genes encoding PCWDEs, the expression profiles of eight such genes (*pelA*, *pelB*, *pelC*, *pelZ*, *pehA*, *pehN*, *pelW*, and *pmeB*) were analysed in the wild type or ∆*eda* mutant strains grown in pyruvate or in pyruvate supplemented with PGA. The results revealed that the expression levels of all eight genes in ∆*eda* were similar to that of the wild type when grown in pyruvate (Figure 7a–h). However, in the presence of pyruvate plus PGA, compared to pyruvate alone, the expression levels of four enzymes (*pelA*, *pelC*, *pehN*, *pelW*) were significantly up‐regulated in the wild type (>5‐fold) (Figure 7a,b,d,e), while six enzymes (*pelA*, *pelC*, *pelZ*, *pehN* [5–10‐fold], and *pelW, pmeB* [>10 fold]) were significantly down‐regulated in ∆*eda* (Figure 7a–f). In particular, *pelW* and *pmeB* were 40‐ and 180‐fold, respectively, down‐regulated in ∆*eda*. Overall, this shows that in the presence of PGA, increased expression of the genes encoding PCWDEs occurs through the action of *eda* but not via control of or through the above regulators, suggesting either direct control by Eda or control through regulators untested in this study.

## 3 DISCUSSION

The genus *Pectobacterium* contains many species, some of which are found on a wide range of host plants. Our previous work showed that host plant extracts can induce expression of the Eda protein, which was found to be required for full virulence of *P. carotovorum* PccS1 (Wang et al., 2018). Eda is part of the ED metabolic pathway that also includes the Edd protein (Figure 1). The ED pathway was shown to be required for full virulence of the animal pathogenic bacteria *E. coli* MG1655 and *V. cholerae* (Chang et al., 2004; Patra et al., 2012). However, in our current study we observed that the *edd* gene is not present in the PccS1 genome (Figure 2a), raising questions about the role of the ED pathway, Eda and Edd in the virulence of *Pectobacterium* spp. We chose to further investigate this pathway using *P. atrosepticum* strains SCRI1043 and SCRI1039, in which we found both genes to be present.

To ensure that PccS1, SCRI1043, and SCRI1039 are not atypical in *eda*/*edd* gene content within the genus, we undertook a phylogenetic study of 14 strains from seven *Pectobacterium* species, using *Dickeya dadantii* 3937 (a close relative of *Pectobacterium*) as an outlier. Our phylogenetic analysis, based on 31 housekeeping genes, indicated that all the *P. atrosepticum*, *P. wasabiae*, and *P. parmentieri* strains tested (Clade 2) possess a complete ED pathway (Figure 2b). In our study, *P. parmentieri* SCC3193, WPP163, and RNS08.42.1A were clustered together in line with the results of the complete‐genome‐based phylogeny of Zhang et al. (2016), although in their paper they were referred to as *P. wasabiae* before being renamed *P. parmentieri* (Khayi et al., 2016). Thus, this supports our phylogenetic analysis based on 31 housekeeping genes in *Pectobacterium* species.

We then searched a further 65 *Pectobacterium* genomes and it was confirmed that of all *Pectobacterium* strains tested, the *edd* gene was only found in these three species (data not shown). Of the three clades identified, species in Clades 1 and 3 are associated with a wide host range (dicots and monocots) and monocots, respectively, while species in Clade 2 have narrow host ranges on dicot plants only (*P. atrosepticum* and *P. parmentieri* on potato and *P. wasabiae* on Japanese horseradish) (Khayi et al., 2016). Although not confirmed, it is interesting to speculate that the presence/absence of the *edd* gene, and therefore the complete ED pathway, may have a role to play in this host range differential.

Sugar metabolism provides various intermediates and energy for bacterial growth with the EMP, PP, and ED pathways involved in metabolism in gram‐negative bacteria (Conway, 1992). *Pectobacterium* produces large quantities of enzymes that utilize pectin from plant cell walls, with breakdown products that ultimately lead into these pathways (Toth et al., 2006). KDPG is one of the breakdown products in pectin metabolism, which feeds into the ED pathway (Figure 1). Because *P. atrosepticum* contains both the *eda* and *edd* genes, we used strains SCRI1043 and SCRI1039 to elucidate the function of the ED pathway. First, we showed that the pathway was functional in Pba but not in Pcc by showing a reduction in the growth of Δ*edd* on gluconate (the primary carbon source of the ED pathway) compared to the wild‐type strain (Figure 2c). Also, by transferring the *edd* gene from Pba to Pcc we could restore this phenotype in PccS1, that is, gluconate utilization occurs specifically via this pathway and the pathway is dysfunctional in PccS1 due to the absence of the *edd* gene.

To demonstrate whether the ED pathway is required for full virulence in *P. atrosepticum*, after previously finding that the key gene (*eda*) of the ED pathway is important in PccS1 virulence (Wang et al., 2018), we undertook tuber assays and showed that the ∆*eda* exhibited reduced virulence compared to the wild‐type strain, while ∆*edd* was unaffected (Figure 3a). To then ensure that the lack of virulence was not due to a general growth defect, we confirmed that both mutants were able to grow in LB and MM containing glucose (Figure 3b,c). The *eda* result was confirmed in whole plant tests where ∆*eda* exhibited a clear reduction in bacterial numbers during infection compared to the wild‐type strain (Figure 4a,b).

Overall, this suggests that there is a conserved role for Eda in pathogenesis within the *Pectobacterium* genus, but this role does not require the ED pathway, instead pointing to a different mechanism(s) through which Eda operates. We showed previously that the impaired *eda* in PccS1 was affected in its ability to grow on pectin agar medium (Wang et al., 2018). While this was also the case in Pba, we went further by showing that the lack of growth was due to an inability of ∆*eda* to utilize GA and PGA, and that ∆*edd* was unaffected in growth on these substrates (Figure 5a–d). Neither mutant was affected in its ability to grow on cellulase plates (Figure S1a,b). This then prompted an examination of the effect of ∆*eda* on the expression of several transcriptional regulators of pectin degradation enzymes and virulence (*kdgR*, *hexA*, *hexR*, and *rsmA*), all of which affect PCWDE production (Charkowski et al., 2012). *kdgR* is a repressor that binds to 2‐keto‐3‐deoxygluconate (KDG), an intermediate upstream of KDPG in the pectin degradation pathway, thereby negatively regulating the expression of genes involved in pectin degradation (*pelA*, *pelB*, *pelC*, and *pelE*) and catabolism (*kdgT*, *ogl*, and *kdul*‐*kdgF*), and in pectinase secretion (*outT*) in *Dickeya dadantii* (previously *Erwinia chrysanthemi*) (Nasser et al., 1994). In human enteric pathogens, KdgR regulates gluconate metabolism (*E. coli*) and contributes to fitness (*Salmonella enterica*) (George et al., 2016; Pouyssegur & Stoeber, 1974). The *hexA* gene represses the production of PCWDEs, the quorum‐sensing signal molecule OHHL, and virulence in soft rot bacteria but also negatively regulates transcription of a second regulator, RsmB (Mukherjee et al., 2000). Analyses using comparative genomics and experimental approaches showed that HexR is a global regulator and serves as a repressor/activator to control carbon metabolism (Leyn et al., 2011). *hexR* is situated adjacent to the *zwf*‐*eda* operon in *Pectobacterium* strains PccS1 and SCRI1043 (Figure 1a), which is concordant with the findings in *Pseudomonas putida* (Kim et al., 2008). HexR controls the ED pathway in *Pseudomonas fluorescens* (Campilongo et al., 2017).

In the present work, the mRNA levels of all these four regulators were unchanged in the mutant ∆*eda* compared with the wild‐type SCRI1039 in MM supplemented with PGA (Figure 6), suggesting that Eda may influence virulence without involvement of these transcriptional regulators. Whether or not KDPG accumulates in the *eda* mutant still needs further investigation in the presence of PGA, as rapid accumulation of intercellular KDPG was reported to be bacteriostatic in an *eda* mutant of *E. coli* (Fuhrman et al., 1998). However, genes encoding enzymes involved in pectin degradation (*pelA*, *pelC*, *pelZ*, *pehN*, and *pelW*) were significantly induced in the wild type and repressed in the Δ*eda* mutant in the presence of PGA but not pyruvate (Figure 7a–h). Therefore, Eda controls expression of genes involved in pectin degradation at the transcriptional level and not through the main regulators of these enzymes, with a lack of Eda resulting in defective utilization of products (GA and PGA) from pectin degradation (Figure 5c,d) and reduced pectinase (but not cellulase) activity on agar plates (Figures 5a,b and S1a,b).

In summary, our results reveal that the aldolase Eda plays a vital role in pectin degradation, and therefore virulence, with a mutation in *eda* preventing full breakdown of pectin and causing a feedback inhibition of pectinases in *P. atrosepticum* (Chang et al., 2004; Patra et al., 2012). However, the ED pathway appears not to be involved in *Pectobacterium* virulence, unlike in some animal pathogens where gluconate breakdown is necessary for virulence. The presence of a complete ED pathway in some species of *Pectobacterium* requires further investigation but may play an important role(s) in other environments, in host range or in the later stages of infection.

## 4 EXPERIMENTAL PREOCEDURES

### 4.1 Strains, media, and growth conditions

Bacterial strains and plasmids are listed in Table 1. *P. carotovorum* PccS1 and *P. atrosepticum* SCRI1039 were used as wild‐type strains for Pcc and Pba, respectively. *Pectobacterium* strains were maintained in LB medium (Wang et al., 2018) or MM supplemented with appropriate carbon sources (Jiang et al., 2017). Kanamycin (50 μg/ml), streptomycin (50 μg/ml), gentamycin (25 μg/ml), and ampicillin (100 μg/ml) were added as required. Prior to all experiments the bacteria were grown overnight in LB at 28 °C.

**TABLE 1:** Bacterial strains and plasmids used in this study

Bacterial strains or plasmid	Relative genotype or characteristic(s)^a^	Reference or source
***Pectobacterium carotovorum* strains**		
PccS1	Rif^r^, wild‐type *P. carotovorum*	Wang et al. (2018)
WT(pBBR)	Rif^r^, Gm^r^, PccS1 wild type with empty pBBR	This study
PccS1(*edd* ^Pba^)	Rif^r^, Gm^r^, PccS1 wild type with pBBR‐*edd* ^Pba^	This study
***P. atrosepticum* strains**		
SCRI1039	Wild‐type *P. atrosepticum*	JHI collection
∆*eda*	SCRI1039 *eda* deletion mutant	This study
WT(pGemT)	Amp^r^, SCRI1039 wild type with empty pGemT	This study
∆*eda*(pGemT)	Amp^r^, SCRI1039 ∆*eda* with empty pGemT	This study
∆*eda*(pEda)	Amp^r^, SCRI1039 ∆*eda* complemented with pGemT‐*eda*	This study
∆*edd*	SCRI1039 *edd* deletion mutant	This study
***Escherichia coli* strains**		
DH10B	F^–^, *mcrA* Δ(*mrr*‐hsdRMS‐*mcrBC*) φ80 *lacZ*ΔM15 Δ*lacX*74 *recA*1 *endA*1 *araD*139 Δ*(ara, leu)*7,697 *galU galK* λ^–^ *rpsL nupG tonA*	Life Technologies, Inc.
S17‐1	Strep^r^, λ pir lysogen of S17‐1	TakaRa
CC118	Host for pKNG101‐based plasmids	JHI collection
HH26	Mobilizing strain for conjugal transfer	JHI collection
**Plasmids**		
pKNG101	Strep^r^, allelic exchange vector, *sacB*, mobRK2, oriR6K	JHI collection
pBluscript‐II KS^+^	Amp^r^, high copy cloning vector, multiple cloning site in *lacZ*’	JHI collection
pBBR1‐MCS5	Gm^r^, broad host range vector	Kovach et al. (1995)
pGem‐T Easy	Amp^r^, cloning vector	Promega
pBBR‐*edd* ^a^	Gm^r^, pBBR with *edd* operon from Pba SCRI1039	This study
pBlu‐*eda*	Amp^r^, pBluscript with two *eda* flanking fragments	This study
pKNG‐*eda*	Strep^r^, pKNG101 with two *eda* flanking fragments	This study
pBlu‐*edd*	Amp^r^, pBluscript with two *edd* flanking fragments	This study
pKNG‐*edd*	Strep^r^, pKNG101 with two *edd* flanking fragments	This study
pGem‐T‐*eda*	Amp^r^, pGem‐T with *eda* operon fragment	This study

^a^ Rif^r^, rifampicin resistance; Gm^r^, gentamycin resistance_;_ Strep^r^, streptomycin resistance; Amp^r^, ampicillin resistance. JHI, The James Hutton Institute, Dundee, UK.

### 4.2 Construction of plasmids and strains

To introduce the *edd* gene from *P. atrosepticum* into *P. carotovorum*, primers (Pba_edd‐F/Pba_edd‐R) were used to construct plasmid pBBR‐*edd* ^Pba^ containing an *edd* fragment from *P. atrosepticum*. The plasmid pBBR‐*edd* ^Pba^ was then introduced into *P. carotovorum* PccS1 by conjugation. Deletion mutants of genes (*edd* and *eda*) in *P. atrosepticum* SCRI1039 were constructed by homologous recombination as described previously (Coulthurst et al., 2008). The plasmids used in this study are detailed in Table 1. All mutants were checked by PCR and sequencing (data not shown). The primers used to construct and verify mutants are listed in Table S1.

### 4.3 Bacterial growth curves in different media

Bacteria were cultured at 28 °C overnight in LB medium. Cultures were collected by centrifugation at 4,000 × *g* for 15 min at room temperature and resuspended at 10^8^ cfu/ml in 10 mM MgSO_4_. LB and MM plus 0.2% carbon source (glucose, pyruvate, gluconate, GA, and/or PGA) were used to assess growth conditions. For growth in 96‐well plates, 20 µl of culture and 180 µl of medium were added to each well. The plates were covered with lids, shaken lightly to mix, and incubated at 28 °C. Plates were assessed after incubating for 2, 24, 36, 48, 72, 96, and 120 hr to measure the OD_600_ values using a plate reader (Promega). Each strain had four replicates and three experiments were carried out.

### 4.4 RNA extraction

An overnight culture of bacteria was inoculated into fresh medium (1:50) and grown to exponential phase (OD_600_ = 0.4), after which a 5 ml sample was centrifuged at 10,000 × *g* for 5 min at 4 °C. A total of 20 µl lysozyme (100 mg/ml) was added to the centrifuged samples and vortexed vigorously. RNA was extracted by following the Qiagen mini Plant RNA extraction handbook and genomic DNA was removed using a DNase I kit (Qiagen). RNA was then quantified at 260 nm using a NanoDrop DE‐ND‐100 spectrophotometer (Thermo Fisher Scientific). The purity and integrity of RNA were monitored by loading a sample onto a 1% agarose gel.

### 4.5 Real‐time PCR analysis

The cDNA was synthesized using the GoScript Reverse Transcription System (Promega). Briefly, a total of 1 µg RNA was primed with random decamers and reverse transcribed with the qScript RT enzyme. To analyse the expression differences of the genes, RT‐qPCR analysis was performed using a PerfeCTa SYBR Green Fast Mix Kit (Quanta) on a StepOne Real‐Time PCR System (Applied Biosystems). The primers listed in Table S1 were designed by the online tool Primer3 web v. 4.0.0 based on the *P. atrosepticum* SCRI1043 genome sequence in NCBI. The ratio of gene expression was normalized to the level of expression of the housekeeping gene *recA* (Takle et al., 2007). Three independent experiments were performed.

### 4.6 Plant cell wall‐degrading enzyme activity assays

Polygalacturonase and cellulase activity was measured as previous described (Andro et al., 1984; Gilkes et al., 1984). Overnight bacterial cultures were grown to 10^4^ cfu/ml. Samples of 10 μl aliquots were applied to the testing plates and incubated at 28 °C for 72 hr. For the polygalacturonase assay, plates were developed using 7.5% copper acetate for 1–2 hr. Cellulase activity was indicated using 0.2% (wt/vol) Congo red for 15–20 min and then washed using 1 M NaCl and 1 M HCl. The haloes around the colonies were measured. At least three plates were used for each assay, and the experiments were repeated at least three times with at least three replicates.

### 4.7 Potato virulence assay

Potato tubers (cv. Maris Piper) were used to perform the virulence assays of *P. atrosepticum* SCRI1039 wild type and mutants. Briefly, tubers were surface sterilized using 5% bleach and chopped with a sterile knife into 7 mm thick slices. A sterilized cork borer (5 mm diameter) was used to make a 5 mm deep well in the centre of each slice. A total of 50 µl of bacterial suspension (5 × 10^5^ cfu) was added into each well and 10 mM MgSO_4_ was inoculated instead of bacteria as a negative control. At least six potato slices obtained from six different potato tubers were tested for each bacterial sample. The tuber slices were placed onto a sterilized Petri dish and incubated at 22 °C in a moist chamber for 3 days and then the rotted tissues were weighed. The entire experiment was repeated on at least three separate occasions.

### 4.8 Colonization in planta

The colonization in planta assay was performed on the stems of potato (cv. Estima) plants. The stems were stab‐inoculated with 10^5^ cfu bacterial cells in 10 μl of 10 mM MgSO_4_ and the inoculation sites were covered with Vaseline. The plants were maintained at 22 °C. Infected stems were cut at 0, 1, 3, 5, and 7 days after inoculation (dai), weighed and ground in the presence of 10 ml Ringer's buffer (per litre 1.2 g NaCl; 0.62 g sodium lactate; 60 mg KCl, 40 mg CaCl_2_; pH 6.5). The cfu per gram of plant tissue was quantified by dilution plating on crystal violet pectate (CVP) agar plates. Photographs of the plants were taken 3 dai.

### 4.9 Phylogenetic analysis

To analyse the evolution of the ED pathway, phylogeny inference was conducted on 13 strains of *Pectobacterium* spp. The data set was limited to the 12 complete *Pectobacterium* genomes available in the PATRIC database (as listed in Table S2) at the time of study (Gillespie et al., 2011), together with one *Pectobacterium* (PccS1) genome sequenced by our group (unpublished data). The *P. atrosepticum* SCRI1043 (NC_004547) genome was used as a reference and the genome of *D. dadantii* 3937 was set as an outgroup.

The protein sequences of 31 housekeeping genes (listed in Table S3) were extracted and saved in FASTA format. The alignments were conducted with the MAFFT online software and quality checked in TOPALi v. 2.5 (Milne et al., 2004). The alignment columns where an amino acid only appeared in a single sequence were deleted manually. The data sets were concatenated using Sequence Matrix (Vaidya et al., 2011) and the concatenated sequences (7,960 amino acids) were then exported for further analysis.

Phylogenetic trees were estimated with the maximum‐likelihood (ML) method IQ‐TREE using the W‐IQ‐TREE web service (Nguyen et al., 2015). To simplify the analysis, we used Model Finder to find the best‐fit model to conduct the following analysis (models for each partition are listed in Table S3). An ultrafast bootstrap analysis was performed with 1,000 replicates. Bootstrap values from the ML analyses were used for adding statistical support onto congruent nodes of the trees drawn with Dendroscope 3 (Huson & Scornavacca, 2012).

### 4.10 Statistical analysis

All the phenotypic data were analysed using the IBM SPSS Statistics v. 20 program and *p* values were calculated using Duncan's multiple range test.

## Supporting information


**FIGURE S1** Mutants Δ*eda* and Δ*edd* of Pba SCRI1039 have no effect on cellulase activity. (a) The activities of cellulase in Pba SCRI1039 wild type, ∆*eda*, and ∆*edd* were carried out on agar plates. (b) Diameters of haloes around the colonies were calculated and statistically analysed. The *y* axis represents means of percentage compare to the wild type (%) ± *SE* from three independent experiments. NS, no significant difference (*p* < .05; Duncan’s multiple range test)

**TABLE S1** Primers for gene modification and real‐time PCR used in this study

**TABLE S2** Characteristics of *Pectobacterium* and *Dickeya* (outgroup) genomes used in this study

**TABLE S3** Details of 31 housekeeping genes used in this study for the development of a phylogenetic tree of *Pectobacterium* strains

## Data Availability

The data that support the findings of this study are available from the corresponding author upon reasonable request.
