# Supplementary figures and images for: Pectobacterium atrosepticum KDPG aldolase, Eda, participates in the Entner–Doudoroff pathway and independently inhibits expression of virulence determinants
Source: Mol Plant Pathol. 2020 Dec 10;22(2):271–83. doi: 10.1111/mpp.13025 (PMC7814964; doi:10.1111/mpp.13025)

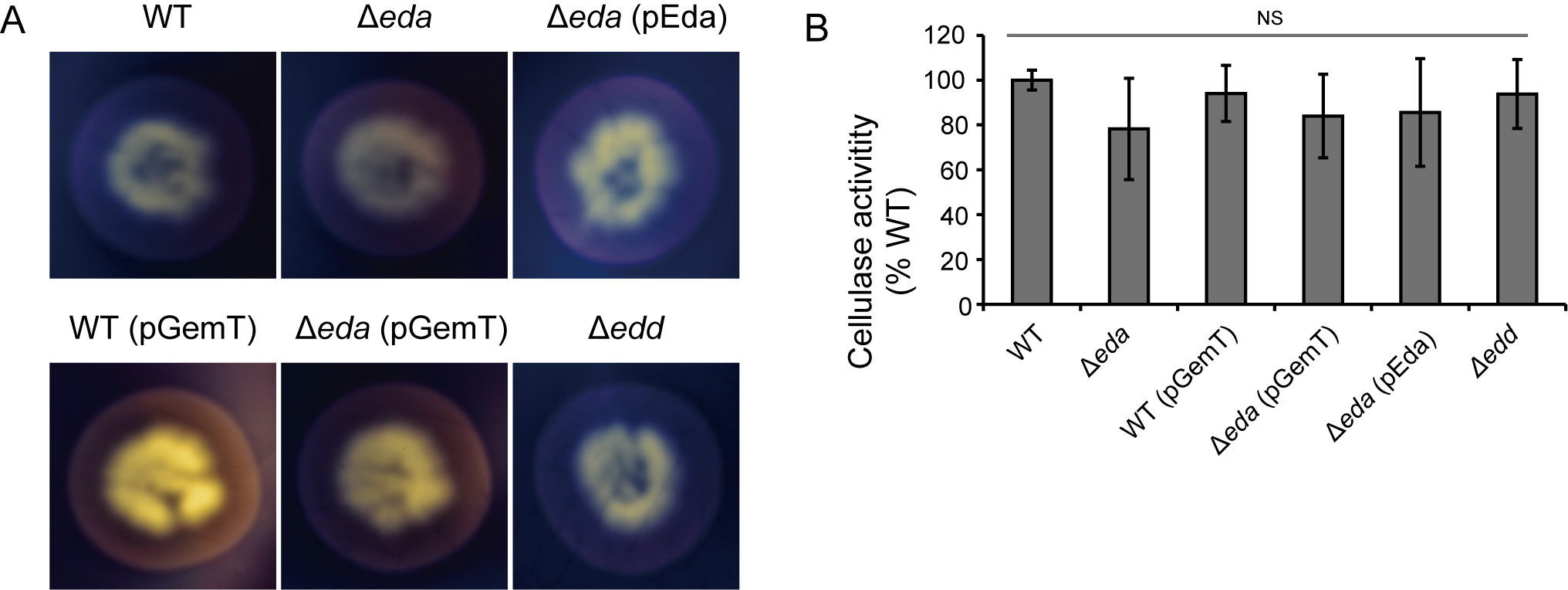

Supplement: Supplementary file 1 — FIGURE S1 Mutants Δeda and Δedd of Pba SCRI1039 have no effect on cellulase activity. (a) The activities of cellulase in Pba SCRI1039 wild type, ∆eda, and ∆edd were carried out on agar plates. (b) Diameters of haloes around the colonies were calculated and statistically analysed. The y axis represents means of percentage compare to the wild type (%) ± SE from three independent experiments. NS, no significant difference (p < .05; Duncan’s multiple range test) [file MPP-22-271-s001.tif]
