# Supplementary material for: Pectobacterium atrosepticum KDPG aldolase, Eda, participates in the Entner–Doudoroff pathway and independently inhibits expression of virulence determinants
Source: Mol Plant Pathol. 2020 Dec 10;22(2):271–83. doi: 10.1111/mpp.13025 (PMC7814964; doi:10.1111/mpp.13025)
Supplement: Supplementary file 2 — TABLE S1 Primers for gene modification and real‐time PCR used in this study [file MPP-22-271-s002.docx]

**Table S1** Primers for plasmid construction, gene modification and quantitative real-time PCR used in this study.

| **Primer** | **Sequence (5’-3’)^a^** | **Application** |
| --- | --- | --- |
| **Gene modification** | | |
| *edd*-F | CGGAATTCACTATCCATCACGCTAAGGC(*Eco*RI) | To amplify a 2500 bp fragment including *edd* gene from Pba SCRI1039 |
| *edd*-R | GCTCTAGATTACTTATCGTCGTCATCCTTGTAATCCGCAGATTCCTGTGGCGAAT (XbaI) |  |
| *eda*-1F | AAATCTAGAGGATGAAGGCGTGGAAATCC (*Xba*I) | To amplify a 500 bp *eda* 5’ flank fragment |
| *eda*-1R | AAAGGATCCCAAAAATTATCTCCAGTCAT (*Bam*HI) |  |
| *eda*-2F | AAAGGATCCGTTTGTCGGTTACTGAATGA (*Bam*HI) | To amplify a 500 bp *eda* 3’ flank fragment |
| *eda*-2R | AAAGGGCCCCCTCACGATACCGGCATGGT (*Apa*I) |  |
| *edd*-1F | AAATCTAGAAGATATTACTATCCATCACG (*Xba*I) | To amplify a 500 bp *edd* 5’ flank fragment |
| *edd*-1R | AAAGGATCCGCTATGCCCCATGTGTTTAA (*Bam*HI) |  |
| *edd*-2F | AAAGGATCCCTACGCACGTTTTAAGCACAA (*Bam*HI) | To amplify a 500 bp *edd* 3’ flank fragment |
| *edd*-2R | AAAGGGCCCAGCAGTAGTCGGCATAACAA (*Apa*I) |  |
| *eda*-CF | CGAAGAAGTCGGAATTGAAG | To amplify a 1664 bp fragment containing the *eda* promoter region |
| *eda*-CR | TCATTCAGTAACCGACAAAC |  |
| *edd*-mR | CAATCCTGGTTTGCACGTCT | To confirm the *edd* knock-out mutant |
| **Quantitative real-time PCR (qPCR)** | | |
| *recA-*F(qPCR) | AGTGGCAGCACCGTTCAAG | JHI collection |
| *recA-*R(qPCR) | CACCCAGATCAACCAGCTCA |  |
| *kdgR*-F(qPCR) | ATTCCGTGTCGTCCGTTTTA | JHI collection |
| *kdgR*-R(qPCR) | GACATAGCCCAGGGATTTCA |  |
| *hexA-*F(qPCR) | CCAGTGCAAATCGACCAGTA | JHI collection |
| *hexA-*R(qPCR) | AGCTCCTTTCCGATGAGTTG |  |
| *hexR-*F(qPCR) | TCGCGACCTTAGCCAAATG | JHI collection |
| *hexR-*R(qPCR) | CGCTGTCATCTTCTTCCACG |  |
| *rsmA*-F(qPCR) | ATGATCGGCGATGAGGTAAC | This study |
| *rsmA*-R(qPCR) | CTTCACGGTGGACAGAAACC |  |
| *pelA*-F(qPCR) | GGAGGTTATGCCACCACTGA | JHI collection |
| *pelA*-R(qPCR) | TTGATCAGCGCATCTTCATT |  |
| *pelB*-F(qPCR) | ACTACACCGGACCGTCTCAC | JHI collection |
| *pelB*-R(qPCR) | CTCAGGTTGCTTAGCCCAAC |  |
| *pelC*-F(qPCR) | GGGGTTACGCTACCACTGAC | JHI collection |
| *pelC*-R(qPCR) | CTGGCCGCAGATATTGTTTT |  |
| *pelZ*-F(qPCR) | GCAGCACCAGAGTTGAAAGG | JHI collection |
| *pelZ*-R(qPCR) | GCCAACCTCGAAAACGATAA |  |
| *pelW*-F(qPCR) | TGGGTTGCTGCACTTGATAG | JHI collection |
| *pelW*-R(qPCR) | GCGTTCTTAGCCAATTCTGC |  |
| *pmeB*-F(qPCR) | CACGCTCCCGGACTATTTTA | JHI collection |
| *pmeB*-R(qPCR) | CCCGGCAGCACTTTAATATC |  |
| *pehA*-F(qPCR) | TTCACGAACAGTGAGCGAAC | JHI collection |
| *pehA*-R(qPCR) | CGCTGAGAAAGACGGAAGAG |  |

^a^ Restriction digestion enzyme sites are underlined.
