# Supplementary material for: Pectobacterium atrosepticum KDPG aldolase, Eda, participates in the Entner–Doudoroff pathway and independently inhibits expression of virulence determinants
Source: Mol Plant Pathol. 2020 Dec 10;22(2):271–83. doi: 10.1111/mpp.13025 (PMC7814964; doi:10.1111/mpp.13025)
Supplement: Supplementary file 3 — TABLE S2 Characteristics of Pectobacterium and Dickeya (outgroup) genomes used in this study [file MPP-22-271-s003.docx]

**Table S2** Characteristics of *Pectobacterium* and *Dickeya* (outgroup) genomes used in this study.

| Strain | Accession No. | Host plant | Country/year |
| --- | --- | --- | --- |
| *P. aroidearum* PC1 | NC_012917.1 | *Ornithogalum dubium* | Israel/2004 |
| *P. aroidearum* PccS1 | Unpublished | *Zantedischia elliotiana* | China/2007 |
| *P. carotovorum* PCC21 | NC_018525.1 | *B. rapa* subsp *pekinensis* | Korea/2012 |
| *P. brasilience* BC1 | NZ_CP009769.1 | *B. rapa* subsp *pekinensis* | China/2002 |
| *P. brasilience* SX309 | NZ_CP020350.1 | *Cucumis sativus.* | China/2015 |
| *P. odoriferum* BC S7 | CP009678.1 | *B. rapa* subsp *pekinensis* | China/2007 |
| *P. atrosepticum* SCRI1043 | NC_004547.2 | *S. tuberosum.* | UK/1985 |
| *P. wasabiae* CFBP3304 | NZ_CP015750.1 | *Eutrema wasabi* | Japan/1987 |
| *P. parmentieri* RNS08.42 | NZ_CP015749.1 | *S. tuberosum* | France/2008 |
| *P. parmentieri* SCC3193 | NC_017845.1 | *S. tuberosum* | Finland/1980 |
| *P. parmentieri* WPP163 | NC_013421.1 | *S. tuberosum* | USA/2004 |
| *D. dadantii* 3937 | NC_014500.1 | *Saintpaulia ionantha* | France/1977 |
