# Supplementary material for: Pectobacterium atrosepticum KDPG aldolase, Eda, participates in the Entner–Doudoroff pathway and independently inhibits expression of virulence determinants
Source: Mol Plant Pathol. 2020 Dec 10;22(2):271–83. doi: 10.1111/mpp.13025 (PMC7814964; doi:10.1111/mpp.13025)
Supplement: Supplementary file 4 — TABLE S3 Details of 31 housekeeping genes used in this study for the development of a phylogenetic tree of Pectobacterium strains [file MPP-22-271-s004.docx]

**Table S3** Details of 31 housekeeping genes used in this study for the development of a phylogenetic tree of *Pectobacterium* strains.

| **No** | **Name** | **Length (AA)** | **Best-fit model according to BIC** |
| --- | --- | --- | --- |
| 1 | *dnaG* | 619 | JTT+G4 |
| 2 | *frr* | 198 | LG |
| 3 | *infC* | 188 | LG+I |
| 4 | *nusA* | 517 | LG+G4 |
| 5 | *pgk* | 410 | JTT+I |
| 6 | *pyrG* | 550 | LG+I |
| 7 | *rplA* | 234 | LG |
| 8 | *rplB* | 280 | JTTDCMut+I |
| 9 | *rplC* | 213 | WAG |
| 10 | *rplD* | 207 | LG+I |
| 11 | *rplE* | 190 | LG |
| 12 | *rplF* | 183 | HIVb+I |
| 13 | *rplK* | 142 | WAG+R2 |
| 14 | *rplL* | 127 | LG |
| 15 | *rplM* | 148 | LG |
| 16 | *rplN* | 174 | cpREV |
| 17 | *rplP* | 140 | LG |
| 18 | *rplS* | 120 | LG |
| 19 | *rplT* | 124 | LG |
| 20 | *rpmA* | 89 | Dayhoff |
| 21 | *rpoB* | 1346 | LG |
| 22 | *rpsB* | 246 | JTT+I |
| 23 | *rpsC* | 260 | HIVb |
| 24 | *rpsE* | 169 | WAG |
| 25 | *rpsI* | 131 | HIVb+I |
| 26 | *rpsJ* | 115 | cpREV |
| 27 | *rpsK* | 130 | cpREV |
| 28 | *rpsM* | 121 | cpREV |
| 29 | *rpsS* | 93 | rtREV |
| 30 | *smpB* | 196 | JTT |
| 31 | *tsf* | 300 | LG+R2 |
